# Secondary Structure Adopted by the Gly-Gly-X Repetitive Regions of Dragline Spider Silk

**DOI:** 10.3390/ijms17122023

**Published:** 2016-12-02

**Authors:** Geoffrey M. Gray, Arjan van der Vaart, Chengchen Guo, Justin Jones, David Onofrei, Brian R. Cherry, Randolph V. Lewis, Jeffery L. Yarger, Gregory P. Holland

**Affiliations:** 1 Department of Chemistry, University of South Florida, 4202 East Fowler Avenue CHE 205, Tampa, FL 33620-9998, USA; gmgray2@mail.usf.edu (G.M.G.); avandervaart@usf.edu (A.v.d.V.); 2 School of Molecular Sciences and the Magnetic Resonance Research Center, Arizona State University, Tempe, AZ 85287-1604, USA; daniel_guo1226@hotmail.com (C.G.); brian.r.cherry@asu.edu (B.R.C.); jyarger@gmail.com (J.L.Y.); 3 Department of Biology and Synthetic Biomanufacturing Center, Utah State University, 650 East 1600 North, North Logan, UT 84341, USA; justin.a.jones@usu.edu (J.J.); randy.lewis@usu.edu (R.V.L.); 4 Department of Chemistry and Biochemistry, San Diego State University, 5500 Campanile Drive, San Diego, CA 92182-1030, USA; romanianknight@gmail.com

**Keywords:** spider silk, NMR, solid-state NMR, molecular dynamics, secondary structure

## Abstract

Solid-state NMR and molecular dynamics (MD) simulations are presented to help elucidate the molecular secondary structure of poly(Gly-Gly-X), which is one of the most common structural repetitive motifs found in orb-weaving dragline spider silk proteins. The combination of NMR and computational experiments provides insight into the molecular secondary structure of poly(Gly-Gly-X) segments and provides further support that these regions are disordered and primarily non-β-sheet. Furthermore, the combination of NMR and MD simulations illustrate the possibility for several secondary structural elements in the poly(Gly-Gly-X) regions of dragline silks, including β-turns, 3_10_-helicies, and coil structures with a negligible population of α-helix observed.

## 1. Introduction

Dragline spider silks have been extensively studied with the long-term goal often being biomimicry [1,2,3]. Dragline spider silks are protein-based biopolymers and understanding the proteins’ primary and secondary structures are critical steps in the goal of reproducing synthetic versions of this extraordinary fiber [4]. The technology and ability to determine primary sequences through DNA analysis has provided numerous amino acid sequences for a large number of dragline silks as well as the diversity of other silks that spiders produce [5,6]. Hence, the next step is to determine the molecular secondary structure and dynamics of these sequenced proteins in spider dragline silk. Protein structural elucidation experimental tools such as nuclear magnetic resonance (NMR) spectroscopy and X-ray Diffraction (XRD) have been extensively used to probe the secondary structures of the proteins that make-up spider dragline silk [7,8,9,10,11,12,13,14,15]. They have provided many insights into the molecular structure and organization of the silk proteins. However, a complete picture of the structure and dynamics within spider dragline silk is still lacking due to the complex and amorphous nature of the biopolymer. The goal of determining a comprehensive protein secondary structure for spider dragline silk protein-based biopolymers is aided by molecular dynamics (MD) simulations, which can play a critical role in connecting experimental restraints with potentially plausible molecular structures [16,17,18]. Much of the complexities in both the structure and dynamics of biopolymers such as spider’s dragline silk will require a synergistic effort between computational/theoretical biophysics and experimental structural biology to obtain a true molecular level structural and dynamic understanding [19]. This is a first effort on the part of the authors to combine recent solid-state NMR results and MD simulations to help elucidate the secondary structures found in the poly(Gly-Gly-X) of orb-weaving spider dragline silk.

Major ampullate spider silk (dragline) is a protein-rich biopolymer that is commonly made up of repetitive amino acid segments (or motifs) from two proteins, major ampullate spidroin 1 (MaSp1) and major ampullate spidroin 2 (MaSp2) in orb-weaving spiders [20]. Common repetitive segments or motifs include poly(Ala), poly(Gly-Ala), poly(Gly-Gly-X), and poly(Gly-Pro-Gly-X-X) [4]. The general picture that has emerged to describe major ampullate spider silk is that the poly(Ala) and flanking poly(Gly-Ala) segments form nanocrystalline β-sheet structures and the rest is an amorphous glycine-rich flexible linking region, where poly(Gly-Gly-X) is the common motif found in MaSp1 and poly(Gly-Pro-Gly-X-X) is the common motif found in MaSp2 [18]. Previous NMR studies have shown that poly(Gly-Pro-Gly-X-X) found in MaSp2 forms type II β-turn structures [21]. Additionally, solid-state NMR has provided evidence that poly(Gly-Gly-X) found in MaSp1 forms 3_1_-helical structures similar to polyglycine II [22,23,24,25,26,27,28,29]. The poly(Gly-Gly-X) sequence is also found in minor ampullate and flagelliform (capture spiral) silk. This sequence is of particular interest as the X residue is always from a restricted set of amino acids [30,31] and is frequently in the same order in each protein. In major and minor ampullate silks they are Leu, Tyr, Ala, and Gln and in flagelliform they are Ala, Val, Ser, and Tyr. In this paper, we combine experimental solid-state NMR results that focus on the X-residues and molecular dynamics simulations to better understand the molecular secondary structure of poly(Gly-Gly-X) found in MaSp1 which will provide a starting point for understanding the structure of this motif in the other silks.

## 2. Results

### 2.1. Solid-State NMR

**Figure 1:**
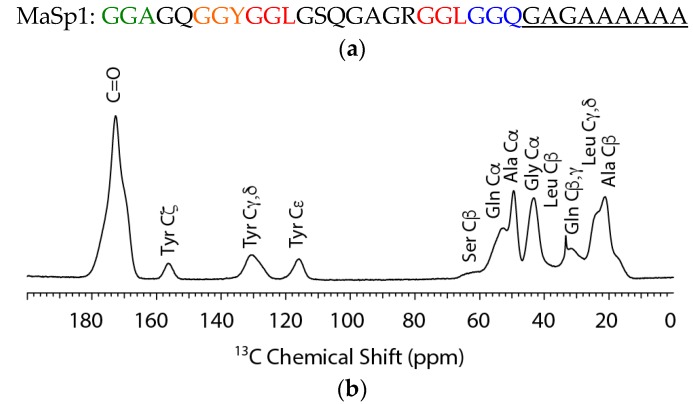
(**a**) Consensus primary amino acid sequence for *N. clavipes* MaSp1. Gly-Gly-X regions where X = A, Y, L and Q are indicated in green, orange, red, and blue; and (**b**) ^1^H→^13^C cross polarization magic angle spinning (CP-MAS) NMR spectrum of ^13^C-labeled *N. clavipes* dragline silk.

The consensus primary amino acid sequence for MaSp1 along with the ^13^C cross polarization magic angle spinning (CP-MAS) NMR spectrum of ^13^C-labeled *N. clavipes* spider dragline silk is shown in Figure 1a,b, respectively. The *N. clavipes* silk is a MaSp1-rich silk with a low MaSp2 content (~80:20, MaSp1:MaSp2). Thus, when investigating this silk it is the MaSp1 protein that is primarily characterized. However, it should be noted that although the *N. clavipes* dragline silk is primarily MaSp1, minor contributions from amino acids present in the MaSp2 protein cannot be discounted. The contribution from MaSp2 amino acids in non-Gly-Gly-X motifs is believed to be mostly negligible for the X amino acids, since Leu is entirely absent from MaSp2 and Tyr is present in the same Gly-Gly-X motif in MaSp2 [6]. Gln is present in a non-Gly-Gly-X Gln-Gln motif in MaSp2 and could contribute to a minor extent. The spider dragline silk is ^13^C enriched at Ala, Gly, Leu, Gln, and Tyr. The Ala methyl, Cβ, resonance has been shown in previous studies to be heterogeneous with a minimum of two-components at 17.4 and 20.9 ppm that has been assigned to Ala present in a disordered 3_1_-helix similar to polyglycine II and ordered nanocrystalline β-sheet structures, respectively [29]. The Ala in 3_1_-helical structures have been ascribed to Ala located in the repetitive Gly-Gly-X motif while, the Ala in β-sheet structures are located in the poly(Ala) and flanking poly(Gly-Ala) motifs in the primary amino acid sequence. By ^13^C labeling the common X amino acids (Gln, Tyr, Leu) found in Gly-Gly-X, we have been able to further probe the secondary structure of this disordered domain.

The ^13^C isotope enrichment permits 2D ^13^C–^13^C correlation experiments with dipolar assisted rotational resonance (DARR) to extract the conformation dependent ^13^C chemical shifts for the various amino acids that are found in the Gly-Gly-X motif. This 2D method is particularly useful for extracting all ^13^C chemical shifts for each site including the CO chemical shifts that are completely overlapped in the 1D ^13^C CP-MAS spectrum (see Figure 1). The ^13^C chemical shifts are tabulated in Table 1. The results for Ala and Gly are similar to previously reported solid-state NMR studies where components for β-sheet and 3_1_-helix were observed with nearly identical chemical shifts [29]. For the other ^13^C labeled X amino acids including Gln and Tyr, the chemical shifts indicate that these amino acids are present in non-β-sheet conformations; however, the shifts do not match α-helical structures and shifts similar to those observed for Ala in a model 3_1_-helix for the most part (see Table 1) [22,32,33,34,35]. Specifically, the observed shifts for Cα and CO sites shift to the β-sheet side of random coil (to lower ppm) and the Cβ sites are close to random coil. This is similar to the observations observed for Ala in the model 3_1_-helical structure, but are also similar to β-turn chemical shift trends depending on the amino acid position in the turn [36]. Lastly, for Leu, the ^13^C chemical shift trends most closely match the random coil conformation. Overall, the solid-state NMR results illustrate that the structure of the Gly-Gly-X motif is not β-sheet or α-helix and is best interpreted as a disordered structure with evidence for 3_1_-helix, β-turn, and/or random coil conformations.

**Figure 2:**
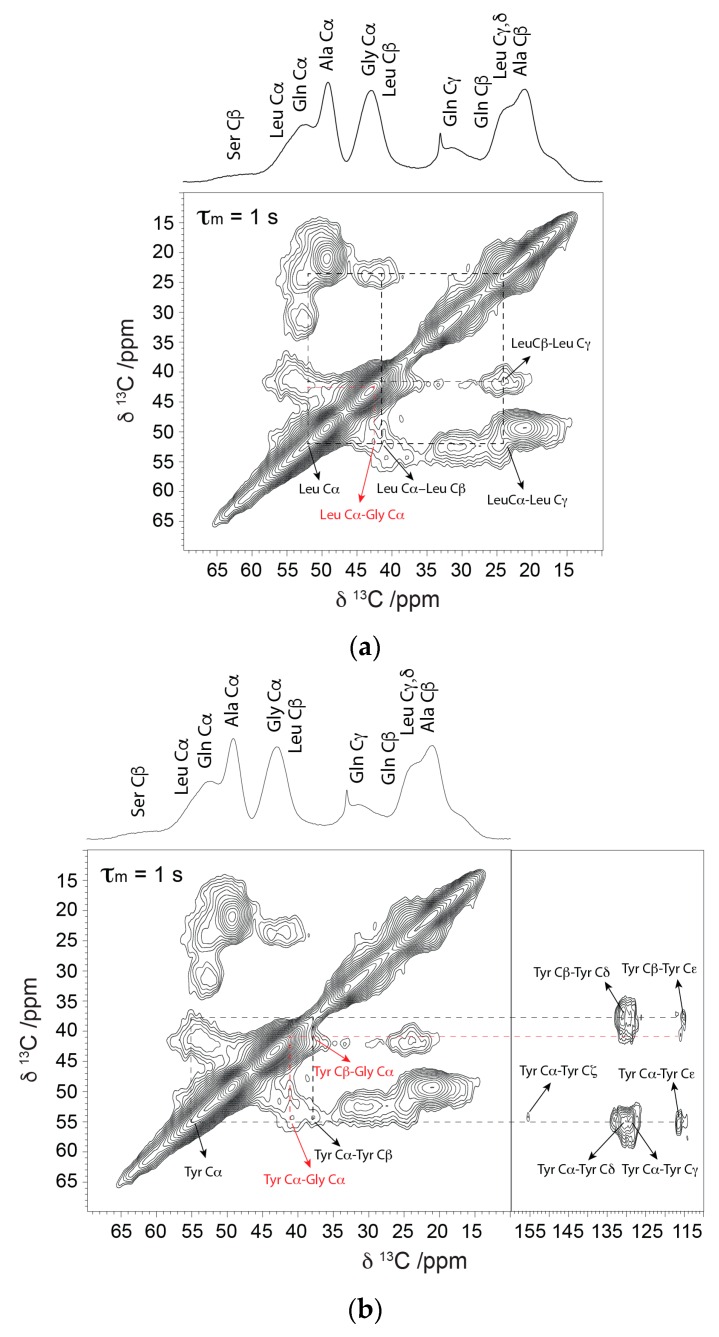
^13^C–^13^C correlation spectrum collected with a long dipolar assisted rotational resonance (DARR) mixing period of 1 s. Short range (intra-residue) and long range (inter-residue) dipolar contacts are indicated with dashed black lines. Long range dipolar contacts for (**a**) Leu-Gly and (**b**) Tyr-Gly present in Gly-Gly-X repeats are indicated in red.

The ^13^C–^13^C correlation method with long (1 s) DARR mixing permits observation of long range contacts between adjacent amino acids. This allows one to identify the location of amino acids in different motifs. For Gln, Tyr, and Leu, long-range contacts to Gly are observed at 42.1, 41.2, and 42.5 ppm, respectively, which is consistent with these amino acids present in the Gly-rich Gly-Gly-X motif (see Figure 2). Importantly, the Gly correlations observed are consistent with Gly present in 3_1_-helix (41.4–42.5) indicating that the common X-amino acids in Gly-Gly-X exhibit correlations consistent with this conformation. However, as discussed above, although the shift trends agree with the 3_1_-helical structure for the most part, the shifts for the X amino acids show some inconsistencies and closely overlap with β-turn and coil conformations. The MD simulations discussed in the next section help to clarify this ambiguity and assist in characterizing the structure of Gly-Gly-X.

### 2.2. Molecular Dynamics Simulations

**Figure 3:**
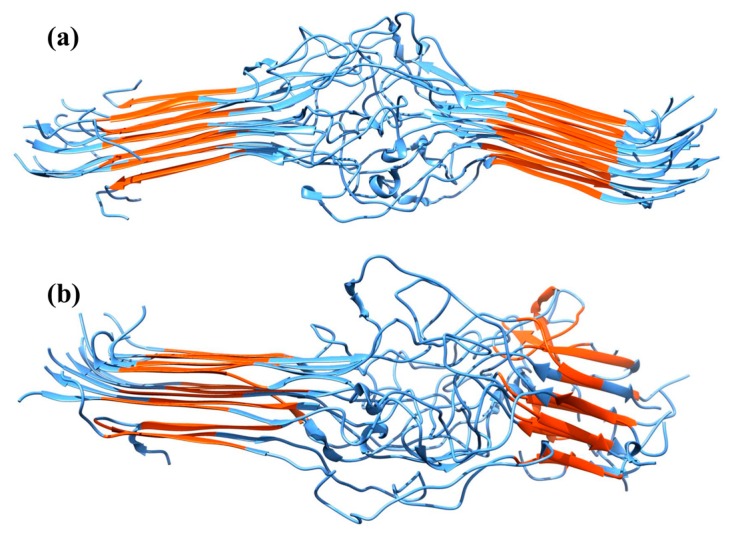
Representative TREX-MD structures at 300 K, with poly(Ala) region in red, rest in blue. (**a**) AP; (**b**) AA system.

Simulations were performed of two MaSp1 mini-fibrils that consisted of three planes of five identical strands. The systems differed in the arrangement of strands: in the anti-parallel/parallel (AP) system, the strands were oriented anti-parallel within the planes and parallel between the planes, while in the anti-parallel/anti-parallel (AA) system, the strands were arranged in an anti-parallel manner within the planes and between the planes. Representative temperature replica exchange molecular dynamics (TREX-MD) structures at 300 K are shown in Figure 3. In all systems, the poly(Ala) regions were in the β-sheet configuration, but the length of the β-sheets varied among the systems. AP had the longest β-sheets, with more residues in this conformation than AA. Root mean square deviations (RMSD) of the backbone atomic positions were calculated using the averaged system as a reference (Table 2). AP showed lower average RMSD values than AA, indicating less structural variation of AP. RMSD values for the spacer region were similar to the overall RMSD for AP and AA, indicating significantly higher mobility of the spacer regions. In both systems, a bend between the two poly(Ala) regions was observed (Figure 3, Table 2). This bending was decomposed into in-plane bending (within the plane of the sheets) and out-of-plane bending (out of the plane of the sheets). The AP bending was small for all three angles; AA had larger bending angles, particularly for out-of-plane bending. The lower bending angle for the AP system suggests that it can be packed more efficiently into larger structures as opposed to the AA system.

**Figure 4:**
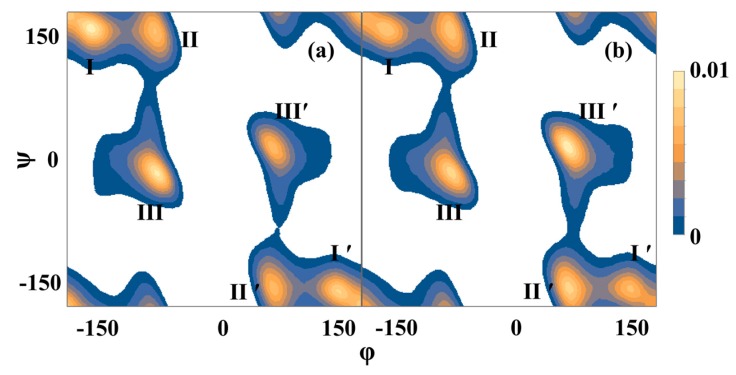
Ramachandran plots for the spacer region (residues 82–107) for (**a**) AP and (**b**) AA simulations. Data is shown for the 300 K replicas. Three basins (and their mirror images, indicated by primes) were found. Basin I corresponds to β-turns and β-sheets, basin II corresponds to β-turns, 3_1_-helices, and coils, and III corresponds to 3_10_-helices and α-helices.

The secondary structure of the spacer region in the TREX simulations is shown in Table 2, and the Ramachandran plot for the spacer region is shown in Figure 4. The spacer regions were rich in β-turns and coils, but a significant fraction of β-sheets was found as well. A large fraction of the poly(Ala) β-sheets extended into the first 5 residues of the spacer region. Typically only a few isolated β-sheets were found in the center of the spacer regions; these generally consisted of a few residues and 2 to 3 strands. This means that the high population of β-sheets stemmed from a continuation of the poly(Ala) β-sheets; apart from these extensions, the spacer had few β-sheets. The spacer was also poor in α-helices, with less than 1% of the spacer residues in α-helical conformation. The low abundance of α-helices and β-sheets in the spacer region is in agreement with the NMR results reported above and in the literature for the Gly-Gly-X region [22,23,24,25,26,27,28,29].

**Figure 5:**
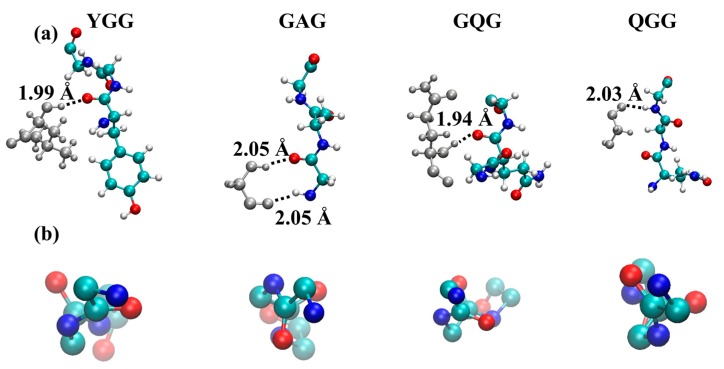
Representative structures of 3_1_-helices in the simulations. (**a**) shows the motifs with hydrogen-bonding partners; while (**b**) shows the backbone atoms along the helical axis. Hydrogen, oxygen, nitrogen, and carbon are indicated in grey, red, blue and green, respectively. Hydrogen bonds with neighboring strands are indicated by dotted lines, and their lengths are given.

Based on NMR chemical shifts, it has previously been reported and also indicated in this study that the spacer region is rich in 3_1_-helices with (φ, ψ) values near (−90°, 150°) [22,23,24,25,26,27,28,29]. We indeed saw a large population of secondary structure elements with these dihedral angles in the simulations, but these were classified as β-turns in our MD analysis. In contrast to the NMR findings, very low 3_1_-helical content was found in the spacer region (~1 per 6 simulation frames). The 3_1_-helices that formed were rich in Gly (Table 3), and were 3 residues in length. Structures of representative 3_1_-helices are shown in Figure 5. The helices formed inter-strand hydrogen bonds, mostly with β-turns. Although NMR chemical shifts of 3_1_-helices show overlap with chemical shifts for β-turns, and the dihedral angles of 3_1_-helices and β-turns overlap, the low occurrence of 3_1_-helices in the simulations likely indicates a force field deficiency in describing Gly-rich areas.

**Figure 6:**
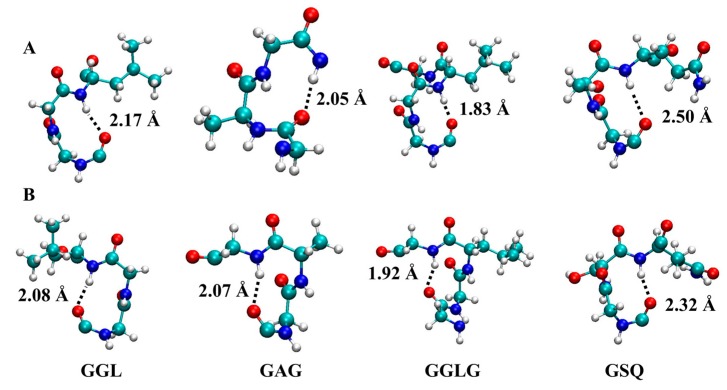
Structure of the most commonly formed 3_10_-helices. Top panels (**A**) show right-handed 3_10_-helices, bottom panels (**B**) show left-handed helices. Hydrogen, oxygen, nitrogen, and carbon are indicated in grey, red, blue and green, respectively. Hydrogen-bond lengths are indicated by dotted lines, and their lengths are given.

In the simulations, 3_10_-helices also formed (4%); mostly consisting of three residues, and sometimes four (~10%). The (φ, ψ) distribution for the 3_10_-helices peaked at (−70°, −25°) and (70°, 25°), which is close to the (−60°, −30°) and (60°, 30°) values for the ideal right and left-handed 3_10_-helix, respectively [37]. The (φ, ψ) dihedral distribution of the 3_10_-helices partly overlapped with the dihedral distribution for β-turns, in particular type I and its mirror image type I’ [37]. β-turns and 3_10_-helices both have i→i + 3 hydrogen bonding patterns, and their chemical shifts largely overlap. There are other structural similarities as well [38,39,40]; in fact, type III β-turns (which are excluded from our β-turn definition) correspond to a 3_10_-helix [37]. In the simulations, the interconversion of β-turns and 3_10_-helices was frequently observed, including β-turns with (φ, ψ) angles that match those of the 3_1_-helix (especially in the higher temperature replicas) although they were short turns only comprised of a few residues and not continuous extended helices. Of interest was the relatively large occurrence of left-handed 3_10_-helices (Table 4). The backbone (φ, ψ) angle distribution of these helices peaked at (70, 25) degrees, which differs from the (−70, −25) degrees of right-handed 3_10_-helices. Left-handed 3_10_-helices are rare in ordinary proteins, and typically involve Gly [37]. The high occurrence of Gly in the MaSp1 spacer region is atypical for proteins; moreover, the absence of tertiary structure in the amorphous spacer region might further contribute to its high formation. Representative structures of the 3_10_-helices are shown in Figure 6.

α-helical motifs were rare; when they occurred, they were on average 2–3 residues longer than the 3_10_-helical motifs. Formation of α-helices occurred only in the central spacer region, except for the (Ala)_n_ α-helices in AA, which were due to refolding of the poly(Ala) region. Since refolding of the poly(Ala) region is unlikely to occur, the AA system is likely not representative of a spider silk mini-fibril. Ser-Gln-Gly (SQG) was present in most of the motifs, and a large percentage of α-helices contained Leu-Gly-Ser (LGS) motifs. Both LGS and SQG sequences also formed 3_10_-helices, suggesting potential interconversion between these structures.

## 3. Discussion

TREX-MD simulations of two MaSp1 mini-fibrils that differed in the arrangement of strands indicated higher stability of the AP system in which the strands were arranged in an anti-parallel manner within and parallel between the planes. The simulations showed that the β-sheets of the poly(Ala) region extend into the first residues of the spacer region. The secondary structure of the remaining spacer region was poor in α-helices and β-sheets, and predominantly consisted of β-turns and coils. The simulations showed very low 3_1_-helical content though, which might point to deficiencies in the force field. A minor fraction of 3_10_-helices was found, with a high occurrence of left-handed 3_10_-helices, which rarely occur in other proteins. It is thought that the high Gly content and the absence of tertiary structure is responsible for the high formation of left-handed 3_10_-helices in the disordered spacer region. Only short 3_1_- and 3_10_-helices were found, with all 3_1_- and most 3_10_-helices consisting of three residues. Conversions between these two structural elements and β-turns were frequently observed. The variation in turns and 3_1_ and 3_10_-helicies appear possible due to the high Gly content and the absence of tertiary structure restraints.

In principle, combining solid-state NMR with MD simulation is a powerful approach for determining the secondary structure for spider dragline silk and the various repetitive motifs that comprise the silk proteins. The solid-state NMR data provided convincing evidence that the Gly-Gly-X motif does not form α-helical or β-sheet structures, with some evidence for the polyglycine II 3_1_-helical conformation. However, some of the observed chemical shifts also overlap with 3_10_-helical, β-turn, and random coil chemical shifts, making the interpretation somewhat ambiguous. The MD simulation provided evidence for the presence of all of these structures, illustrating the disorder of the Gly-Gly-X spacer region and helping with the NMR interpretation. While 3_1_-helical content is currently underestimated in the MD, tuning of the Gly force field parameters and the use of chemical shift restraints in the simulations will improve the accuracy of the simulations. In this way, it is anticipated that combining solid-state NMR and MD will greatly enhance our ability to characterize the conformational structure of the various repetitive motifs that comprise spider and other types of animal silks.

## 4. Materials and Methods

### 4.1. Materials

Mature female *N. clavipes* spiders were fed with tap water and crickets once per week. Spiders were forcibly silked at a speed of 2 cm/s for 1 h every other day. The major ampullate silk (dragline silk) was separated from the minor ampullate silk under an optical microscope (Olympus, Waltham, MA, USA). To prepare isotope enriched dragline silk, the spiders were fed a 200 μL saturated solution of U-[^13^C, ^15^N]-l-alanine, U-[^13^C, ^15^N]-l-leucine, U-[^13^C, ^15^N]-l-glutamine, and U-[^13^C, ^15^N]-l-phenylalanine over four feedings during silk collection. A total of 10 mg of isotope-enriched dragline silk was collected. Isotopes were purchased from Cambridge Isotopes Laboratories, Inc. and used as received.

### 4.2. Solid-State NMR Measurements

Solid-state NMR spectra were collected on a Varian VNMRS 400 MHz spectrometer equipped with a 1.6 mm triple-resonance cross polarization magic angle spinning (CP-MAS) probe operating in triple resonance mode (^1^H/^13^C/^15^N). One-dimensional (1D) ^1^H→^13^C CP-MAS and two-dimensional (2D) ^13^C–^13^C through-space correlation NMR experiments with dipolar-assisted rotational resonance (DARR) experiments [29] were performed at a spinning speed of 35 kHz. The CP condition consisted of a 1.6 μs ^1^H π/2 pulse, followed by a 1.0 ms ramped (6%) ^1^H spin-lock pulse with a radio frequency (rf) field strength of 155 kHz at the ramp maximum and the ^13^C channel matched to the −1 spinning sideband condition (rf field strength of 120 kHz). Typical experimental conditions included a 25 kHz sweep width, and a recycle delay of 3.0 s, with two-pulse phase-modulated (TPPM) ^1^H decoupling applied during acquisition with a rf field strength of 130 kHz. In 2D ^13^C–^13^C through-space correlation experiments, the spectra were collected with 1024 points in the direct dimension, 320 *t*_1_ complex points in the indirect dimension, and 32 scans averages with spectral widths in the direct and indirect dimension of 25 and 35 kHz, respectively. During the DARR mixing period, continuous wave (CW) irradiation was applied on the ^1^H channel at *n* = 1 (*ω*_R_ = *ω*_1_) rotary resonance condition with mixing times (τ_m_) of 50, 150 ms, and 1 s. The 2D spectra were processed with exponential line broadening of 100 Hz in the direct dimension and a Gaussian function of the form *exp*(−(*t*/*gf*)^2^) in the indirect dimension with the constant, *gf*, equal to 0.0025. The ^13^C isotropic chemical shift was indirectly referenced to adamantane (38.56 ppm).

### 4.3. Molecular Dynamics Simulations

Simulations of spider silk mini-fibrils consisting of MaSp1 residues 71–121 with primary sequence GQGAGAAAAA-AGGAGQGGYG-GLGSQGAGRG-GLGGQGAGAA-AAAAAGGAGQ-G were performed. Each strand consisted of two poly(Ala) regions, separated by a spacer region. The individual strands were capped by acetyl and amine groups. The mini-fibrils were constructed from 3 planes of 5 strands, for a total of 15 identical strands. Each of these strands was initially constructed in an extended β-sheet conformation. Two different systems were simulated in which the strands were oriented antiparallel within the planes, and parallel or antiparallel between the planes. These systems are designated AA (antiparallel within planes, antiparallel between planes) and AP (antiparallel within planes, parallel between planes), respectively.

In order to significantly enhance the amount of sampled space, temperature replica exchange molecular dynamics (TREX-MD) [41,42] simulations were performed. In TREX-MD, multiple independent copies of the system (replicas) are run at different temperatures. At regular time intervals, attempts are made to swap coordinates between the replicas with neighboring temperatures. The success of these attempts is based on an energy criterion that preserves detailed balance. In order to equilibrate the replicas at their chosen temperatures, molecular dynamics (MD) simulations were performed. These simulations used distance restraints between the Cα atoms of opposing sheets of poly(Ala) regions; these distances were restrained by a flat bottom potential with a force constant of 20 kcal/(mol Å), active beyond a distance of 6.0 Å. Each system was first heated from 120 to 400 K over a period of 1 ns. Replicas of the system were then cooled to their TREX-MD starting temperature over a period of 1 ns. The temperatures for the TREX-MD simulations were selected from unrestrained TREX-MD trial runs so as to optimize the swapping of replicas. In the trial runs, systems were simulated at temperature intervals of 10 K between 300 and 400 K. The heat capacity was then calculated from these exploratory simulations and the temperature of the phase transition corresponding to the melting of the noncrystalline area was identified (between 350 and 365 K). Because phase transitions represent bottlenecks for replica exchange [43], smaller spacings were used near the phase transition. Swapping was monitored in further trial runs, and extra replicas were inserted where needed. This optimization resulted in 37 common replicas at temperatures of 300, 302, 304, 307, 309, 311, 313, 315, 317, 319, 322, 324, 326, 329, 333, 334.5, 336, 339, 341, 343, 345, 347, 350, 352, 355, 357, 359, 361, 363, 365, 367, 369, 371, 373, 375, 377, and 380 K. Additional replicas were added at 305.5, 320, 327.5, 331, 337, 348.5, and 353 K for AP, and 305.5, 321, 327.5, 331, 337.5, 353.5, and 378.5 K for AA, resulting in a total of 44 replicas for each system.

After temperature equilibration, a 5 ns per replica TREX-MD equilibration was performed, during which the use of positional restraints on the poly(Ala) region was removed. All simulations were performed with the Amber12 GPU [44] code and the Amber99SB [45] force field, using a generalized Born implicit solvent model [46], and Langevin dynamics with a friction coefficient of 5 ps^−1^. Bonds involving hydrogen atoms were constrained using the SHAKE algorithm [47], which permitted the use of a 2 fs timestep. Swapping was attempted every 2 ps for all systems, with an average success rate between 50% and 70% for each replica. Coordinates were saved every 2 ps. After equilibration, an unrestrained production run of 60 ns per replica was performed for AP, and 45 ns for AA, for a total production simulation time of 2.6 and 1.9 μs, respectively. Simulations were run until all replicas had visited all temperatures; this took somewhat longer for the AP system. Secondary structures for all but the 3_1_-helices were calculated using STRIDE [48], which uses the Kabsch and Sander rules [38] with stricter hydrogen bond definitions for assigning α- and 3_10_-helices and β-sheets, and Thornton’s definitions [39,40] for turns. For this analysis, all β-turn types were grouped together, but type III (which equals a 3_10_-helix) was excluded. STRIDE assignments were verified by visual inspections. Since 3_1_-helices are not identified by STRIDE, visual inspections were performed on samples of three consecutive residues, of which at least two residues had |φ| between 70° and 90°, and |ψ| between 140° and 150°; absolute values were chosen to include both right- and left-handed helices. A total of 400 randomly selected structures were visually inspected for the occurrence of 3_1_-helices; the sample statistics were used to calculate the overall occurrence of 3_1_-helices, and boot strapping was used to estimate errors. All structural analyses were performed for the 300 K replicas.

## 5. Conclusions

Solid-state NMR and MD simulations were used in conjunction to illuminate the conformational structure of poly(Gly-Gly-X), one of the most common repetitive motifs found in dragline spider silk proteins. The combination of NMR and MD results provides new insight into the secondary structure of poly(Gly-Gly-X) segments and provides further support that these regions are disordered and primarily non-β-sheet. Further, the combination of NMR and MD simulations illustrate the possibility for several secondary structural domains in the poly(Gly-Gly-X) regions of dragline silks including β-turns, 3_10_-helicies, and coil structures with an insignificant population of α-helix observed. These solid-state NMR results and MD simulations highlight the complexity of this common spider silk protein motif. It is envisioned that this combined NMR experimental and MD computational method will be powerful moving forward for elucidating the conformational structure and hierarchical organization of other silk motifs that remain under determined.

## Figures and Tables

**Table 1:** ^13^C NMR isotropic chemical shifts in ppm from tetramethylsilane (TMS) for *N. clavipes* dragline silk, random coil, and polypeptides with defined secondary structures.

Residue	*N. clavipes*	α-Helix	β-Sheet	Random Coil	3_1_-helix ^a^
Gly Cα	43.2	46.0	43.2–44.3	43.4	41.4–42.5
Gly CO	169.7	174.9	168.4–169.7	173.2	
Ala Cα	49.3	52.3–52.8	48.2–49.3	50.8	48.9
Ala Cβ	50.3	14.8–16.0	19.9–20.7	17.4	17.4
	17.4				
	20.9				
Ala CO	172.8	176.2–176.8	171.6–172.2	176.1	174.6
	174.5				
Gln CO	172.6	175.4–175.9	171.9–172.2	174.3	–
Gln Cα	52.7	56.4–57.0	51.0–51.4	54.0	–
Gln Cβ	25.8	25.6–26.3	29.0–29.9	27.7	–
Gln Cγ	30.6	29.7–29.8	29.7–29.9	32.0	–
Gln Cδ	176.6	–	–	178.8	–
Tyr CO	172.7	176.7	169.7	174.2	–
Tyr Cα	55.1	54.8–58.6	52.1	56.2	–
Tyr Cβ	37.7	36.1	39.3	37.1	–
Tyr Cγ	128.6	129.7	128.0	128.9	–
Tyr Cδ	130.9	129.7	128.0	131.6	–
Tyr Cε	116.1	116.1	115.0	116.5	–
Tyr Cζ	156.2	154.2	155.2	155.6	–
Leu CO	176.0	175.7	170.5–171.3	175.9	–
Leu Cα	53.8	55.7	50.5–51.2	53.4	–
Leu Cβ	41.6	39.5	43.3	40.7	–
Leu Cγ	24.0	–	–	25.2	–
Leu Cδ	23.3	24.4	24.9	24.3	–

^a^ (AGG)_10_ model peptide with (φ, ψ) values near (−90°, 150°).

**Table 2:** Structural data of 300 K replica. Root-mean-square deviation (RMSD) in Å and angles in degrees. Secondary structural elements in the spacer regions (residues 82–107) are given as a percentage of all spacer residues; π-helices and bridges are not reported.

Region	Property	AP	AA
Overall	RMSD	9.2 ± 1.0	11.5 ± 0.8
	Total bending angle	13.2 ± 7.5	55.5 ± 18.7
	In-plane bending angle	6.5 ± 0.7	16.0 ± 5.6
	Out-of-plane bending angle	5.4 ± 4.2	31.3 ± 18.9
Spacer region	RMSD	9.6 ± 1.5	11.4 ± 3.4
	% 3_10_-helix	4.1	4.3
	% 3_1_-helix	0.1	0.1
	% β-turn	30.6	35.6
	% Coil	32.5	33.4
	% β-sheet	26.4	18.9
	% α-helix	0.7	0.9

**Table 3:** Sequence composition of 3_1_**-**helices in the 300 K replicas. X indicates a residue with |φ| between 70 and 90 degrees, and |ψ| between 140 and 150 degrees; Y indicates any combination of dihedral angles.

% Residue	AP		AA	
	XX	XYX	XX	XYX
G	60.0 ± 4.9	58.5 ± 3.3	61.5 ± 4.8	60.0 ± 3.0
A	9.6 ± 4.4	5.2 ± 2.7	12.1 ± 4.8	8.0 ± 3.2
Q	8.5 ± 3.9	12.2 ± 3.5	10.1 ± 4.5	10.0 ± 3.3
L	9.2 ± 4.2	8.0 ± 3.1	7.9 ± 3.7	8.9 ± 3.2
Y	3.7 ± 1.9	6.1 ± 2.7	5.3 ± 2.7	7.3 ± 3.1
R	9.4 ± 4.3	6.6 ± 2.8	4.9 ± 2.5	3.4 ± 1.8
S	3.6 ± 1.7	4.2 ± 2.1	2.6 ± 0.4	3.5 ± 1.9

**Table 4:** 3_10_-helices in the 300 K replicas. %G, %A, and %S indicate the occurrence of Gly, Ala, or Ser, respectively, given as a percentage of all residues in 3_10_-helices. The average length of hydrogen bonds in the 3_10_-helices (in Å) is also shown.

Occurrence	AP			AA		
	Overall	Left	Right	Overall	Left	Right
% G	50.9	41.5	58.5	49.0	53.3	46.7
% A	36.6	47.3	52.7	17.6	67.3	32.7
% S	13.8	21.6	78.4	11.8	46.6	53.4
3_10_ hydrogen bond length	2.1 ± 0.2			2.1 ± 0.2		
